# Preoperative Neutrophil-to-Lymphocyte Ratio Was a Predictor of Overall Survival in Small Renal Cell Carcinoma: An Analysis of 384 Consecutive Patients

**DOI:** 10.1155/2020/8051210

**Published:** 2020-03-06

**Authors:** Hao Zhao, Wang Li, Xiang Le, Zixiang Li, Peng Ge

**Affiliations:** Department of Urology, The Affiliated Hospital of Xuzhou Medical University, Xuzhou, Jiangsu, China

## Abstract

**Objective:**

The aim of this study was to investigate the prognostic significance of the preoperative neutrophil-to-lymphocyte ratio (NLR) in small renal cell carcinoma (sRCC, ≤4 cm).

**Methods:**

This study was approved by the review board (NO.XYFY2019-KL032-01). Between 2007 and 2016, a total of 384 consecutive patients who underwent curative surgery for sRCC at our institution were evaluated. Patients were divided into high NLR and low NLR groups by plotting the NLR receiver operating characteristic curve. The Kaplan–Meier method was utilized to graphically display survivor functions. Univariate and multivariate Cox proportional hazards regression analysis addressed time to overall survival (OS) and cancer-specific survival (CSS).

**Results:**

Of the 384 patients, 264 (68.8%) were males and 120 (31.2%) were females. Median follow-up time after surgical resection was 54 months. One hundred and eighty-seven (48.7%) patients had a high NLR (≥1.97), and the remaining 197 (51.3%) had a low NLR (<1.97). Patients with high NLR were more likely to be aged compared with patients with low NLR (*P*=0.028). High NLR was associated with decreased OS and CSS compared with low NLR (*P*=0.028). High NLR was associated with decreased OS and CSS compared with low NLR (*P*=0.028). High NLR was associated with decreased OS and CSS compared with low NLR (*P*=0.028). High NLR was associated with decreased OS and CSS compared with low NLR (

**Conclusions:**

Elevated preoperative NLR is an independent adverse prognostic factor for OS after surgery with curative intent for sRCC.

## 1. Introduction

There will be an estimated 73,820 new cases and 14,770 deaths from kidney & renal pelvis cancer in the United States in 2019 [1]. Renal cell carcinoma (RCC) accounts for more than 90% of all kidney malignancies [2]. Among these cases, clear cell RCC was the predominant type. Due to the advancements and penetration of modern radiologic imaging techniques, the incidental detection of small renal cell carcinoma (sRCC, ≤4 cm) has been steadily increasing [3].

Although sRCC is believed to have a favorable prognosis, a subset of these carcinomas is associated with aggressive features, including synchronous or metachronous metastasis [4]. Improved understanding of sRCC biology may facilitate patients counseling in prognosis prediction [5]. Plenty of prognostic factors of RCC have been validated. Of those, the TNM stage and nuclear grade are currently widely used; however, they are not entirely reliable [6, 7]. Therefore, some new prognostic factors need to be identified.

Decipherment of the molecular mechanisms underlying renal tumorigenesis allows yielding new diagnostic and prognostic markers. Systemic inflammatory response, of which the neutrophil-lymphocyte ratio (NLR) is often used as an indicator, has been shown to convey a significant influence on tumorigenesis and tumor development [8, 9]. Growing evidence supports that increased preoperative NLR predicts poor outcome in a wealth of cancers, including primary colorectal carcinoma, breast cancer, lung cancer, as well as kidney cancer [10]. More recently, Nunno and colleagues implemented a systematic review and meta-analysis and proposed that higher NLR was negatively correlated to overall survival (OS) and progression-free survival in both metastatic and nonmetastatic patients [11].

However, the issue whether NLR affects oncological outcomes in sRCC remains unclear. Therefore, the present study was intended to investigate the prognostic significance of the preoperative NLR in sRCC.

## 2. Materials and Methods

### 2.1. Patient Population

The study was approved by the ethical committee of the Affiliated Hospital of Xuzhou Medical University (no. XYFY2019-KL032-01). Between 2007 and 2016, a total of 444 consecutive patients who underwent curative surgery including radical or partial nephrectomy for sRCC at our institution were evaluated retrospectively. In the present study, sRCC was defined as the tumor had 4 cm or less on postoperative pathological evaluation. Patients without complete clinicopathologic and follow-up data were excluded from the study. In order to make a homogeneous entity, only pT1N0M0 patients were included. Finally, a total of 60 subjects were eliminated. The remaining 384 patients were included in the study.

The patient's characteristics were extracted from the medical records and pathological reports, and all the data were entered into a database. Pathological stage was assigned according to the American Joint Committee on Cancer (8th edition) [12]. In particular, only patients with complete absolute lymphocyte count and absolute neutrophil count data within 2 weeks before surgery were included in the study [13]. NLR was calculated by dividing the neutrophil measurement by the lymphocyte measurement.

### 2.2. Follow-up

Postoperative follow-up was not standardized. Generally, patients were evaluated quarterly during the first year, semiannually during the next 2 years, and then annually. Examinations included laboratory and imaging studies unless otherwise clinically indicated. The main endpoints were OS and cancer-specific survival (CSS). Cause of death was determined by treating physicians and/or by chart review and was corroborated by death certificates if available. OS was defined as the time from the date of surgery until death due to any cause, and CSS was defined as the time from the date of surgery to a kidney cancer-related death. Surviving patients were censored at the last follow-up.

### 2.3. Statistical Analysis

Associations of categorical variables were assessed using the chi-square test. Receiver operating characteristic (ROC) curve analysis was performed to detect if the NLR had a distinctive feature regarding OS and CSS. The cutoff value was determined by the Youden index, which maximized the vertical distance from the reference line [14]. The Kaplan–Meier method was utilized to assess the survival curve, and the statistical significance was determined by the log-rank test. Univariate and multivariate Cox proportional hazards regression analysis addressed time to cancer-specific and overall mortality. The hazard ration (HR) estimated by Cox analysis was reported as a relative risk with a corresponding 95% confidence interval (CI). Statistical analysis was performed using the Statistical Analysis System version 9.4 (SAS Institute, Cary, NC, USA) or Statistical Package for the Social Sciences 25.0 software (SPSS Inc., Chicago, IL, USA). Two-sided *P* < 0.050 was considered statistically significant.

## 3. Results

### 3.1. Association with Clinical and Pathological Characteristics

A total of 384 patients with sRCC were included in the present study. Among them, 264 (68.8%) were males and 120 (31.2%) were females, with a median age of 57 years (Table 1). Of note, 28 (7.3%) patients presented with other primary tumors (multiple primary neoplasms), synchronously or metachronously. All patients underwent surgical treatment, including 87 cases of open surgery, 297 cases of laparoscopic surgery, 231 cases of radical nephrectomy, and 153 cases of partial nephrectomy.

**Figure 1:**
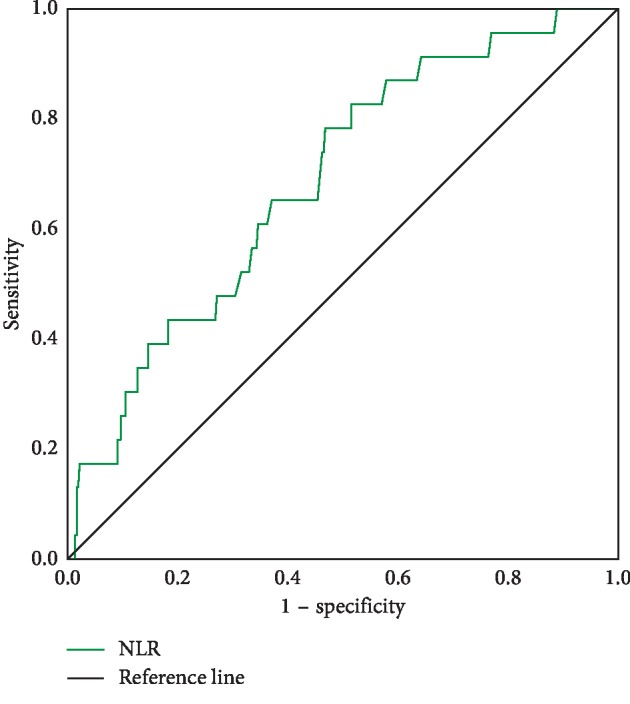
ROC curve analysis for overall survival of NLR.

The median neutrophil count was 3.68 × 10^9^/L, and the median lymphocyte count was 1.89 × 10^9^/L. The median NLR was 1.96. Based on the area under the curve (AUC) for survival in the ROC analysis (Figure 1), the Youden index was applied to determine the optimal cutoff value of 1.97 for NLR. We dichotomized the NLR, i.e., high NLR (≥1.97) versus low NLR (<1.97). Among these patients, 197 (51.3%) had a high NLR and the remaining 187 (48.7%) had a low NLR. As is shown in Table 1, the patients with high NLR were more likely to be aged (≥60, *P* = 0.028).

### 3.2. Overall Survival

**Figure 2:**
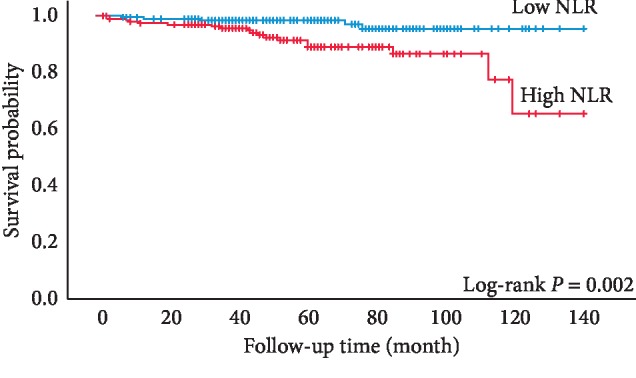
Kaplan–Meier curves for sRCC patients' overall survival categorized by NLR (cutoff value = 1.97).

The median follow-up from the surgery was 54 (range 2–143) months. During the follow-up, overall deaths occurred in 18 (9.6%) patients with high NLR and in 5 (2.5%) with low NLR.  Figure 2 exhibits that patients with high NLR were associated with a higher risk for overall mortality than patients with low NLR (*P*=0.002, log-rank test) (Table 2). The same findings held true for multivariate analysis after being adjusted for the effects of age and multiple primary neoplasms (HR 3.145, 95% CI 1.158–8.545, *P*=0.025, Table 3).

Considering the presence of multiple neoplasms may be a confounding factor, subgroup analysis was undertaken. When we excluded the patients with multiple primary neoplasms and reanalyzed, the results were consistent, i.e., high NLR was associated with decreased OS compared with low NLR (*P*=0.027, log-rank test, [Supplementary-material supplementary-material-1], Supplementary Materials). Moreover, in order to improve the homogeneity of the study, analysis after omitting nonclear cell RCC cases was conducted. The similar result was found (*P*=0.005, log-rank test, [Supplementary-material supplementary-material-1], Supplementary Materials).

### 3.3. Cancer-Specific Survival

**Figure 3:**
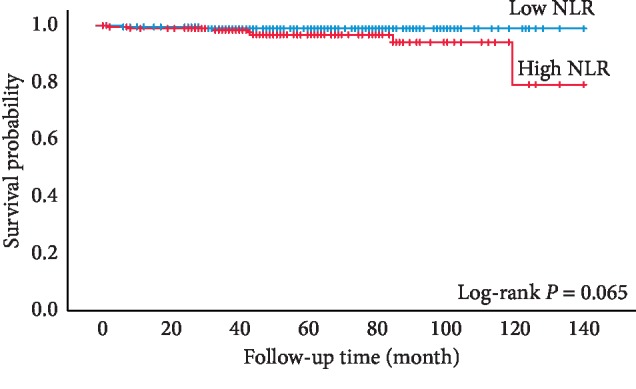
Kaplan–Meier curves for sRCC patients' cancer-specific survival categorized by NLR (cutoff value = 1.97).

During the follow-up, 9 (2.3%) patients died of sRCC. In univariate analysis, high NLR patients had a lower probability for CSS than patients with low NLR; however, this difference was not significant (Figure 3, *P*=0.065, log-rank test) (Table 2). Multivariate analysis demonstrated that presence of multiple primary neoplasms and age were independent prognostic factors associated with cancer-specific mortality (Table 3).

## 4. Discussion

This is a study to assess the prognostic significance of the preoperative NLR in sRCC. The present study showed that patients with high NLR were associated with a higher risk for overall mortality than patients with low NLR.

Increasing evidence suggests that the systematic inflammatory response plays a crucial role in caner development and progression [15]. Biomarkers of systemic inflammatory can be categorized into two indices: a differential blood cell count (neutrophil, monocyte, lymphocyte, and platelet) and concentration of specific serum proteins (albumin, C-reactive protein, and fibrinogen) [16], and these indicators have been proven to be related to the prognosis of patients with a variety of cancers including RCC [10, 11, 13, 17, 18]. NLR has become an intriguing parameter in the prognosis model of patients with RCC due to its advantageous properties such as wide application range, low cost, and easy access in the clinical setting [19].

The exact mechanisms underlying the association between increased NLR and adverse prognosis are complex and remain to be elucidated. One potential mechanism is that the elevated NLR reflects the enhancement of neutrophil-dependent inflammatory response and the decrease in lymphocyte-mediated antitumor immune response [20, 21]. Neutrophils have been shown to secrete several chemokines and cytokines, such as transforming growth factor-beta, interleukin-6, interleukin-8, and so on, and these agents contribute to tumor development, progression, and metastasis [10, 20, 22]. In contrast, lymphocytes are associated with antitumor immunity, and lymphopenia reflects the impaired lymphocyte-dependent immune response. Several studies highlighted the importance of lymphocytes and demonstrated that increasing infiltration of tumors with lymphocytes showed better prognosis in cancer patients [23]. Taken together, NLR may be reflective of the combined prognostic information of these two processes and is stronger than the predicted results alone.

As mentioned earlier, preoperative elevated NLR has been shown to be associated with poor prognosis in patients with RCC in numerous previous studies [11]. However, to the best of our knowledge, there has been no study emphasizing on the prognostic significance of the preoperative NLR in sRCC alone. The results of our current series showed that a high NLR was an independent adverse predictor for OS but not a predictor for CSS. Our findings were consistent with Bazzi et al.'s, who investigated a total of 1970 patients who underwent partial or radical nephrectomy for nonmetastatic RCC, and the results demonstrated that increased preoperative NLR elevation was associated with worse OS, but had no significant association with CSS or relapse-free survival [24]. In another study, Pichler et al. reported similar results [20]. In addition, the findings may be still held true even for advanced RCC. Baum and colleagues [25] retrospectively analyzed the clinicopathologic data of 1871 patients with metastatic clear cell RCC who underwent cytoreductive nephrectomy and found that elevated preoperative NLR may be associated with increased overall mortality. However, in contrast to our negative findings, Byun et al. identified increased NLR to be an independent predictor of recurrence-free survival and CSS (each *P* < 0.05) [13].

It should be pointed out that the definitive explanations for the somewhat contradictory results regarding OS and CSS remain speculative. One probable hypothesis is that NLR level is an alternative marker of systemic inflammation with medical conditions, such as hypertension, diabetes, cardiovascular diseases, renal insufficiency, and kinds of cancers [24]. Thus, the increased overall mortality in the high NLR group is possibly a reflection of patients' comorbidities [20]. In this study, we found that patients with high NLR were more likely to have multiple primary neoplasms and older age, further strengthening this speculation. Jeong et al. [4] demonstrated that age at diagnosis was an independent prognosis predictor after curative surgical treatment in sRCC patients and suggested that older patients should be followed more closely after surgery than younger patients. Another explanation is that the statistical power was weakened by very few cancer-related events. According to Figure 3, high NLR patients had a trend toward a lower probability of CSS, though no significant difference was found.

Previous studies suggest high variability in the optimal NLR cutoff value [26]. Several methods are used in calculating the cutoff value, for example, the ROC curve, mean, tertile, median, and log-rank test. The standard cutoff value remains unknown. In this study, the cutoff value calculated from the Youden index was 1.97, which was close to the median value 1.96. In order to improve the strength of the study, we utilized the median value as cutoff and reanalyzed the data. The conclusion drawn above remained the same (Figures [Supplementary-material supplementary-material-1], [Supplementary-material supplementary-material-1], and [Supplementary-material supplementary-material-1], Supplementary Materials). Further study should emphasize particularly on standardizing cutoff values to facilitate the decisional value in the clinics.

Given the favorable prognosis for sRCC and the low complication rates and procedural morbidity, focal therapy is becoming more commonly utilized, such as cryoablation and radiofrequency ablation, microwave ablation, and irreversible electroporation [27]. The American Urological Association guidelines recommend ablation as an alternative for small renal lesions less than 3 cm [27]. In this study, however, we only included patients who underwent curative surgery including radical or partial nephrectomy. Thus, the prognostic value of NLR for patients who underwent focal therapy is unclear and needs further investigation.

Our study also had some limitations. First and foremost are the limitations inherent to their retrospective nature, requiring further prospective studies to confirm our findings. In the second place, as alluded to earlier, NLR is not a specific disease marker and several conditions can influence its specificity, such as active infection and inflammatory diseases, as well as stress at the time of blood drawing [20, 24], and we did not exclude this part of patients. Therefore, the use and generalizability of NLR as a specific prognostic indicator is impaired [20]. Thirdly, considering the low risk of mortality for patients with sRCC after surgery, our data was limited by an overall short follow-up time, and the statistical power was weakened by very few cancer-related deaths.

## 5. Conclusions

This study assessed the prognostic significance of the preoperative NLR in sRCC. Patients with high NLR were more likely to be older. Elevated preoperative NLR is an independent adverse prognostic factor for OS after surgery with curative intent for sRCC. High NLR patients had a trend toward a lower probability of CSS in univariate analysis, though no significant difference was found. Further studies are needed to verify the present findings.

## Figures and Tables

**Table 1:** Clinical and pathological characteristics in patients with sRCC and low (<1.97) or high (≥1.97) NLR.

	All	Low NLR	High NLR	*P* value
Gender				0.063
Male	264	127 (64.5%)	137 (73.3%)	
Female	120	70 (35.5%)	50 (26.7%)	
Age (years)				**0.028**
<60	223	125 (63.5%)	98 (52.4%)	
≥60	161	72 (36.5%)	89 (47.6%)	
Histologic subtype				0.592
Clear cell RCC	348	177 (89.8%)	171 (91.4%)	
Non-clear-cell RCC	36	20 (10.2%)	16 (8.6%)	
Multiple primary neoplasms				0.087
No	356	187 (94.9%)	169 (90.4%)	
Yes	28	10 (5.1%)	18 (9.6%)	
Type of surgery				0.464
Partial nephrectomy	153	82 (41.6%)	71 (38.0%)	
Radical nephrectomy	231	115 (58.4%)	116 (62.0%)	
Surgical approach				0.169
Laparoscopic surgery	297	158 (80.2%)	139 (74.3%)	
Open surgery	87	39 (19.8%)	48 (25.7%)	
IFN-*α*/IL-2 therapy				0.781
No	296	153 (77.7%)	143 (76.5%)	
Yes	88	44 (22.3%)	44 (23.5%)	

Abbreviation: sRCC, small renal cell carcinoma. RCC, renal cell carcinoma. NLR, neutrophil-to-lymphocyte ratio.

**Table 2:** Univariate Cox proportional hazards regression analysis of clinical and pathological characteristics.

	Overall survival			Cancer-specific survival		
	HR	95% CI	*P* value	HR	95% CI	*P* value
Gender						
Male	1			1		
Female	0.576	0.214–1.553	0.275	1.041	0.260–4.162	0.955
Age (years)						
<60	1			1		
≥60	6.763	2.299–19.891	<**0.001**	11.363	1.421–90.865	**0.022**
Histologic subtype						
Clear cell RCC	1			1		
Non-clear-cell RCC	0.425	0.057–3.159	0.403	1.137	0.142–9.105	0.904
Multiple primary neoplasms						
No	1			1		
Yes	9.398	4.116–21.460	**<0.001**	6.549	1.637–26.192	**0.008**
Type of surgery						
Partial nephrectomy	1			1		
Radical nephrectomy	1.904	0.704–5.148	0.205	1.911	0.394–9.269	0.421
Surgical approach						
Laparoscopic surgery	1			1		
Open surgery	1.676	0.718–3.913	0.232	1.096	0.261–4.602	0.900
IFN-*α*/IL-2 therapy						
No	1			1		
Yes	1.495	0.624–3.583	0.368	1.445	0.353–5.922	0.609
NLR						
<1.97	1			1		
≥1.97	4.164	1.545–11.226	**0.005**	3.950	0.819–19.050	0.087

Abbreviation: NLR, neutrophil-to-lymphocyte ratio. HR, hazard ratio. CI, confidence interval.

**Table 3:** Multivariate regression models.

	Overall survival			Cancer-specific survival		
	HR	95% CI	*P* value	HR	95% CI	*P* value
Age (≥60 years)	5.148	1.738–15.248	0.003	9.791	1.212–79.097	0.032
Multiple primary neoplasms	7.156	3.112–16.451	<0.001	4.827	1.192–19.543	0.027
NLR (≥1.97)	3.145	1.158–8.545	0.025	NA		

Abbreviation: NLR, neutrophil-to-lymphocyte ratio. HR, hazard ratio. CI, confidence interval. NA, not applicable.

## Data Availability

The data used to support the findings of this study are available from the corresponding author upon request.

## Abbreviations

NLR: Neutrophil-to-lymphocyte ratio
sRCC: Small renal cell carcinoma
OS: Overall survival
CSS: Cancer-specific survival
RCC: Renal cell carcinoma
ROC: Receiver operating characteristic
CI: Confidence interval
HR: Hazard ration.

## Supplementary Materials

Supplementary Materials Figure S1: Kaplan-Meier curve for overall survival for patients without multiple primary neoplasms categorized by NLR (cutoff value = 1.97). Figure S2: Kaplan-Meier curve for overall survival for clear cell renal carcinoma patients categorized by NLR (cutoff value = 1.97). Figure S3: Kaplan-Meier curves for sRCC patients' overall survival categorized by NLR (cutoff value = 1.96, *n* = 384). Figure S4: Kaplan-Meier curves for sRCC patients' cancer-specific survival categorized by NLR (cutoff value = 1.96, *n* = 384). Table S1: Multivariate regression models (cutoff value = 1.96, *n* = 384).
