# Supplementary material for: Preoperative Neutrophil-to-Lymphocyte Ratio Was a Predictor of Overall Survival in Small Renal Cell Carcinoma: An Analysis of 384 Consecutive Patients
Source: Biomed Res Int. 2020 Mar 6;2020:8051210. doi: 10.1155/2020/8051210 (PMC7079219; doi:10.1155/2020/8051210)
Supplement: Supplementary Materials — Figure S1: Kaplan-Meier curve for overall survival for patients without multiple primary neoplasms categorized by NLR (cutoff value = 1.97). Figure S2: Kaplan-Meier curve for overall survival for clear cell renal carcinoma patients categorized by NLR (cutoff value = 1.97). Figure S3: Kaplan-Meier curves for sRCC patients' overall survival categorized by NLR (cutoff value = 1.96, n = 384). Figure S4: Kaplan-Meier curves for sRCC patients' cancer-specific survival categorized by NLR (cutoff value = 1.96, n = 384). Table S1: Multivariate regression models (cutoff value = 1.96, n = 384). [file 8051210.f1.pdf]

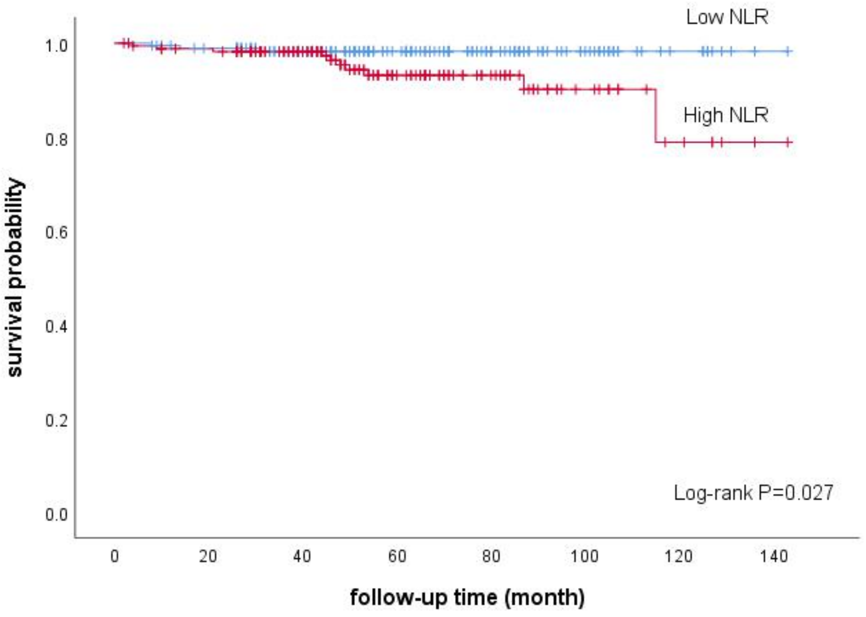

FIGURE S1: Kaplan-Meier curve for overall survival for patients without multiple primary neoplasms categorized by NLR (cutoff value=1.97).

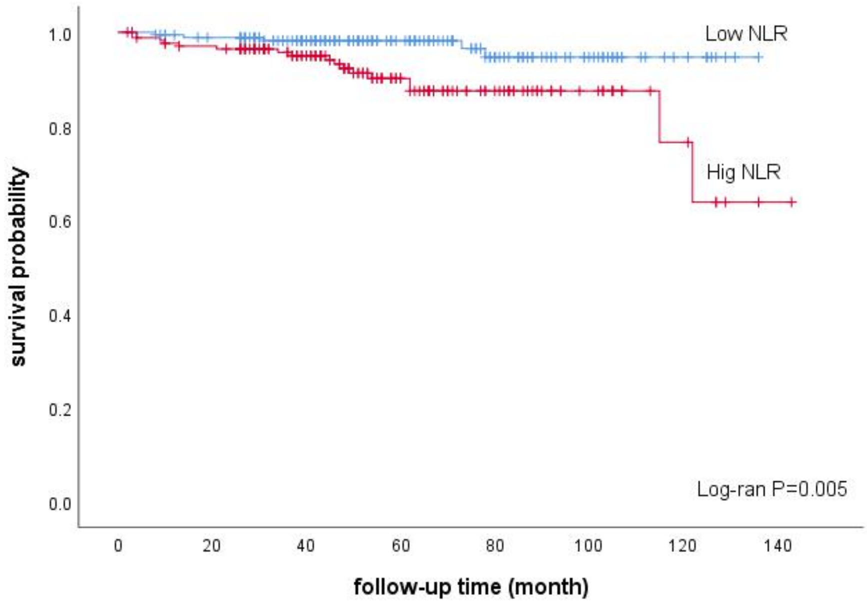

FIGURE S2: Kaplan-Meier curve for overall survival for clear cell renal carcinoma patients categorized by NLR (cutoff value=1.97).

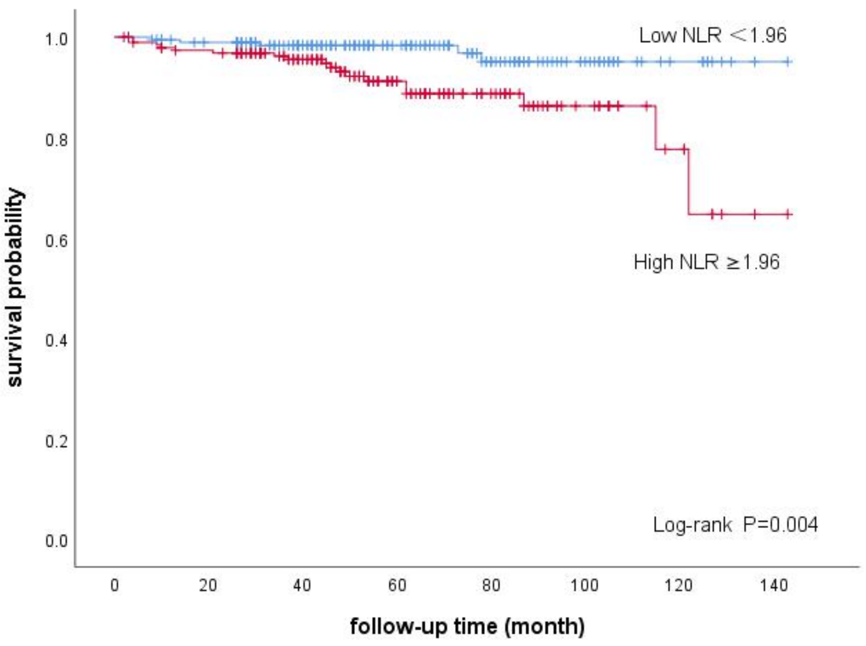

FIGURE S3: Kaplan–Meier curves for sRCC patients' overall survival categorized by NLR (cutoff value=1.96, n=384).

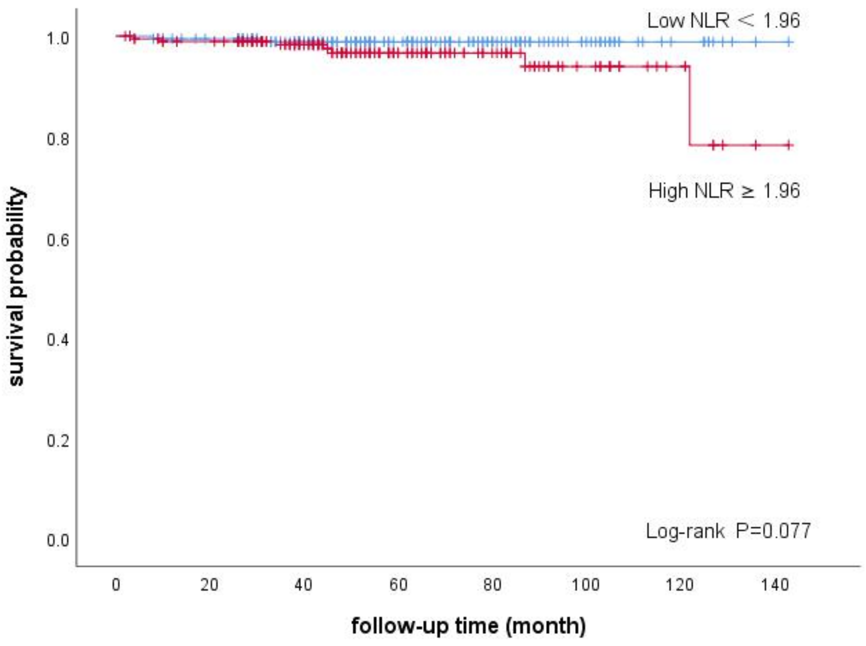

FIGURE S4: Kaplan–Meier curves for sRCC patients cancer-specific survival categorized by NLR (cutoff value=1.96, n=384).

TABLE S1: Multivariate regression models (cutoff value=1.96, n=384).

	Overall survival			Cancer-specific survival		
	HR	95%CI	P value	HR	95%CI	P value
Age (≥60 years)	5.528	1.873-16.317	0.002	9.791	1.212-79.097	0.032
Multiple primary neoplasms	7.021	3.054-16.142	<0.001	4.827	1.192-19.543	0.027
NLR (≥1.96, median)	2.995	1.102-8.138	0.032		NA	

Abbreviation: **NLR**, neutrophil-to-lymphocyte ratio. **HR,** hazard ratio. **CI,** confidence interval. **NA**, not applicable.
